# The Mitigating Effect of Remifentanil Against Sevoflurane-Induced Cardiotoxicity in Human Cardiomyocytes via TRPA1 Channels

**DOI:** 10.3390/jcdd13070339

**Published:** 2026-07-21

**Authors:** Eyüp Aydoğan, İshak Suat Övey, Oğuz Karahan

**Affiliations:** 1 Department of Anesthesiology and Reanimation, Faculty of Medicine, Alanya Alaaddin Keykubat University, 07425 Alanya, Antalya, Türkiye; eyup.aydogan@alanya.edu.tr; 2 Department of Physiology, Faculty of Medicine, Alanya Alaaddin Keykubat University, 07425 Alanya, Antalya, Türkiye; 3 Department of Cardiovascular Surgery, Faculty of Medicine, Alanya Alaaddin Keykubat University, 07425 Alanya, Antalya, Türkiye; oguz.karahan@alanya.edu.tr

**Keywords:** sevoflurane, remifentanil, cardiotoxicity, TRPA1 channels, cardiomyocytes

## Abstract

Introduction: Sevoflurane may exert cytotoxic effects in some cell types, but its impact on human cardiomyocytes and the potential protective role of remifentanil remain unclear. This study aimed to investigate whether remifentanil mitigates sevoflurane-induced apoptotic damage in human cardiomyocytes, with an exploratory focus on TRPA1 calcium channels. Materials and Methods: Human cardiomyocyte cells were exposed to sevoflurane (5.1% for 6 h) and/or remifentanil (2.5 µM for 30 min), with or without the TRPA1 antagonist AP18. Apoptosis, intracellular reactive oxygen species (ROS), mitochondrial depolarisation, caspase-3 and -9 activities, and cytosolic calcium levels were measured. Results: Sevoflurane exposure was associated with increased apoptosis, ROS production, mitochondrial depolarisation, caspase-3 and -9 activities, and intracellular calcium levels compared with the control group. Remifentanil pretreatment appeared to reduce these sevoflurane-related changes. In several parameters, the addition of AP18 partially reversed the effects of remifentanil, suggesting that TRPA1 channels may contribute to these effects. However, findings were not entirely consistent across all measured variables. Conclusions: Remifentanil effectively mitigates sevoflurane-induced apoptotic damage in human cardiomyocytes, likely by modulating oxidative stress and preserving mitochondrial function. The partial reversal by AP18 suggests that TRPA1 channels may contribute to these protective effects, though additional mechanisms are likely involved. Further mechanistic and translational studies are warranted to elucidate the precise signalling pathways and clinical relevance of these observations.

## 1. Introduction

Sevoflurane is a volatile anaesthetic agent widely used in clinical anaesthesia practice. Due to its pharmacokinetic advantages and cardioprotective effects, it is frequently preferred, especially in cardiac surgery. However, studies conducted in recent years have shown that the biological effects of sevoflurane are not always protective and that, under certain conditions, it can cause cytotoxicity and apoptosis via oxidative stress. Piao et al. demonstrated that sevoflurane exposure triggers cell death through DNA damage and parthanatos by increasing intracellular reactive oxygen species (ROS) levels in neuronal cells [1]. Similarly, Mattei et al. reported that sevoflurane induces neurotoxicity in neonatal mice via GSK3β/Drp1-dependent mitochondrial fission and apoptosis [2]. These findings suggest that similar risks may exist for the myocardium, a tissue that is metabolically highly active and sensitive to oxidative damage. Although cardiomyocytes possess endogenous antioxidant defence systems, the increase in ROS that occurs during and after sevoflurane exposure may overwhelm these systems, leading to mitochondrial dysfunction, activation of apoptotic cascades, and loss of functional cardiomyocytes [1].

The precise molecular mechanisms by which sevoflurane-induced oxidative stress initiates apoptosis in cardiomyocytes remain incompletely elucidated. In recent years, transient receptor potential (TRP) ion channels have been recognised as sensors that respond to stimuli such as oxidative stress and inflammation. TRPA1 (Transient Receptor Potential Ankyrin 1), a member of this family, is a calcium channel that can be directly activated by reactive oxygen and carbonyl species. Activation of TRPA1, which is expressed in cardiomyocytes, can disrupt intracellular calcium homeostasis, leading to mitochondrial dysfunction and apoptosis. In this context, the hypothesis that increased ROS induced by sevoflurane may trigger apoptosis in cardiomyocytes by stimulating TRPA1 channels gains strength.

Another agent frequently used together with sevoflurane in anaesthesia practice is remifentanil. Remifentanil, an ultra-short-acting opioid, is preferred especially in major interventions such as cardiac surgery due to its potent analgesic effect and rapid metabolism. Research conducted in recent years shows that remifentanil may have cytoprotective properties in addition to its analgesic effects. Dou et al. (2016) reported that remifentanil administration protects against ischaemia–reperfusion injury in rat cardiomyocytes and that this effect is mediated by δ-opioid receptor activation of PI3K/Akt and ERK signalling pathways [3]. Furthermore, Naldan et al. (2021) demonstrated that remifentanil reduces glutamate toxicity and exhibits neuroprotective effects in rat olfactory bulb neurons [4], and another study showed that TRPA1 antagonists attenuate sevoflurane-induced changes in TRPV1 expression [5]. These findings suggest that remifentanil has protective potential against oxidative stress and excitotoxicity in different cell types. However, the possible modulatory effects of remifentanil on TRPA1 channel activity have not yet been investigated.

The interaction between sevoflurane and remifentanil, which are frequently co-administered during cardiac surgery, may be critical in preventing myocardial damage. Although the hypothesis that remifentanil may protect against the potential pro-apoptotic effects of sevoflurane is supported by the existing literature, the lack of direct investigation of this relationship in the context of TRPA1 channels in human cardiomyocytes remains an important gap. It is thought that remifentanil may inhibit the apoptotic process by either suppressing the sevoflurane-induced increase in ROS or modulating TRPA1-mediated calcium influx.

The primary aim of this study is to investigate the apoptotic activity of sevoflurane in human cardiomyocytes and the possible role of TRPA1 channels in this effect, as well as to evaluate the cytoprotective effects of remifentanil through this pathway. We hypothesise that remifentanil protects cardiomyocytes from apoptosis by reducing ROS levels elevated by sevoflurane exposure or by directly suppressing TRPA1 channel activity. To test this hypothesis, sevoflurane and remifentanil were administered in a human cardiomyocyte culture model; cell viability, apoptosis markers, ROS levels, TRPA1 expression, and signalling pathways associated with activation will be examined. The findings may help determine optimal anaesthetic agent combinations for myocardial protection in the perioperative period and elucidate the role of TRPA1 in this process.

## 2. Materials and Methods

### 2.1. Reagents/Stains

Human Cardiomyocyte Complete Media with Serum, Human Cardiomyocyte Serum-Free Media (Celprogen, Torrance, CA, USA), Trypsin–EDTA, dimethyl sulfoxide, and Dihydrorhodamine-123 (DHR 123) were procured from Sigma-Aldrich (St. Louis, MO, USA). Caspase-3 (AC-DEVD-AMC) and caspase-9 (AC-LEHD-AMC) substrates were procured from Enzo (Lausanne, Switzerland). APO percentage assay with a release buffer was procured from Biocolor (Belfast, Ireland). Fura-2 (AM) fluorescent calcium stain was procured from Calbiochem (Darmstadt, Germany), and Pluronic^®^ F-127 was procured from Biovision (San Francisco, CA, USA). Probenecid and a mitochondrial stain, 5.50, 6.60-tetrachloro-1,10.3.30-tetraethylbenzimidazolylcarbocyanine iodide (JC-1), were procured from Santa Cruz (Dallas, TX, USA).

### 2.2. Cell Culture

**Scheme 1:**
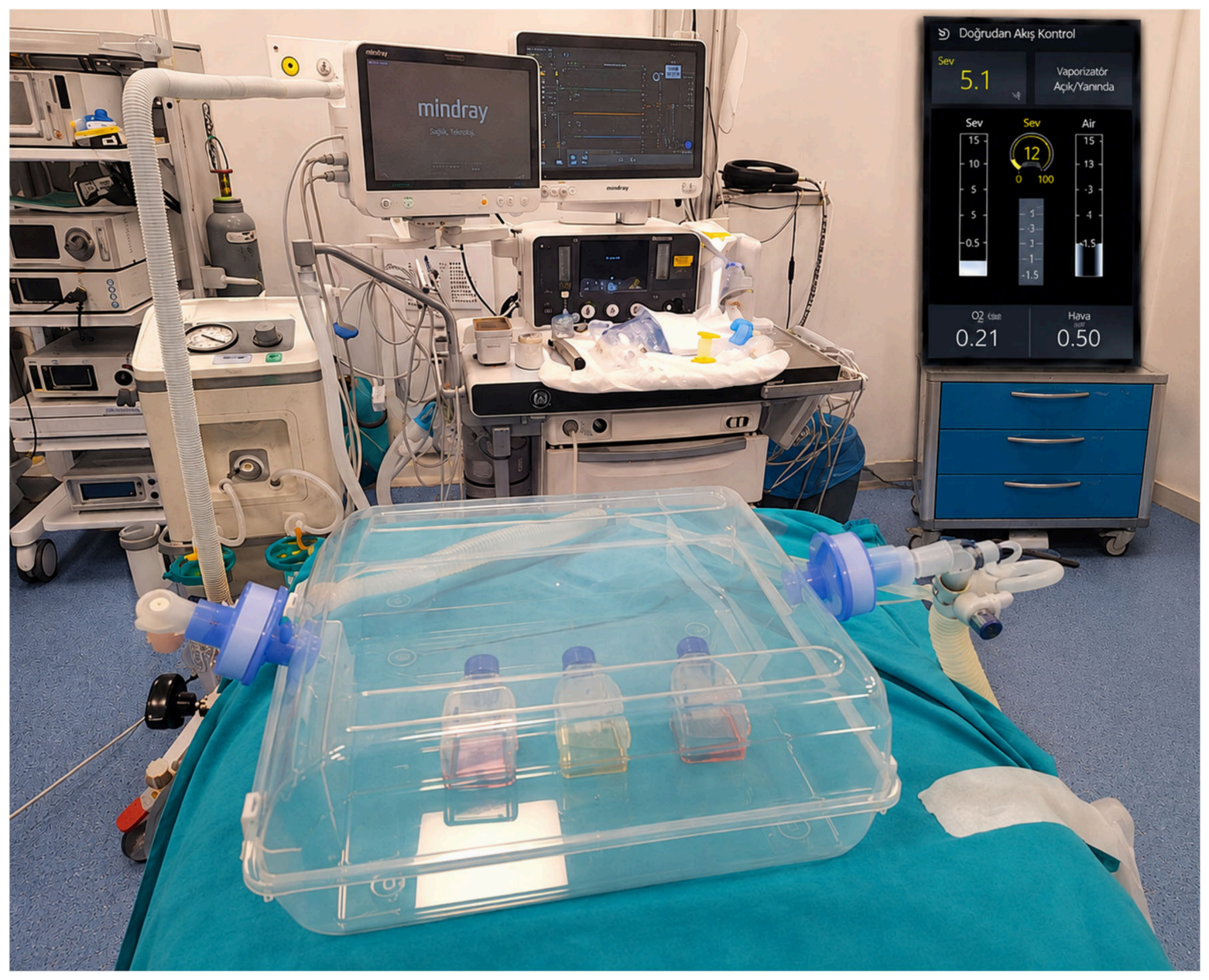
Anaesthesia device and airtight chamber system used during 5.1% sevoflurane exposure of cardiomyocyte cells.

Human cardiomyocyte cells were obtained from Celprogen Inc. (Cat. Number: 36044-15; Celprogen, Torrance, CA, USA). Cells were cultured in Human Cardiomyocyte Complete Media with Serum (the commercial form includes serum and antibiotics). Cultures were maintained in T25 flasks (Human Cardiomyocyte Primary Cell Extracellular Matrix, Cat. Number E36044-15-T25, Celprogen, Torrance, CA, USA) and incubated at 37 °C in a humidified atmosphere with 5% CO_2_. At 75–85% confluence, cells were treated with the compounds described in the experimental groups section. All experimental groups were maintained under identical physical conditions, including temperature (37 ± 0.5 °C), CO_2_, and O_2_ levels, during the exposure period. In addition, a matched control group was maintained in the same sealed chamber system without exposure to sevoflurane. The cells were exposed to 5.1% sevoflurane, 21% oxygen, and 0.04% CO2 for 6 h in a sealed, transparent container with a dual respiratory circuit connected at the inlet and outlet. A gas sampling line was connected to the outlet circuit to continuously monitor the constant volatile anaesthetic concentration (Scheme 1). The incubation case has been heated to the target temperature via an electronically regulated heating device from the base. Temperature changes have been closely monitored and corrected using a laser thermometer. The cell-line-containing chamber was kept closed during the sevoflurane exposure. The gas mixture of 5.1% sevoflurane, 0.04% CO_2_, and 21% O_2_, with a humidity of 60%, was exposed at a flow rate of 0.5 L/min. A Mindray A9 anaesthesia machine from Shenzhen, China, 2025, was used in the study. Following treatment, cells were washed, detached using 0.25% trypsin–EDTA, collected into 15 mL tubes, and centrifuged at 100× *g* for 5 min. After washing with fresh medium, cells were prepared for subsequent experiments.

**Study Groups**

HCCs (Human Cardiomyocyte Cells) were cultured at 37 °C. The cells were divided into seven main groups.

Group 1 (Control): After 75–85% confluence, as in the other groups, HCCs were maintained under similar physical conditions in the operating room, without exposure to sevoflurane or remifentanil.

Group 2 (SEV): HCCs in the group were incubated with 5.1% sevoflurane for 6 h [6].

Group 3 (SEV + AP18): HCCs in the group were incubated with 5.1% sevoflurane for 6 h and then incubated with AP18 (TRPA1 channel antagonist, 0.1 mM, 30 min).

Group 4 (REM): HCCs were incubated with 2.5 µM remifentanil for 30 min [7].

Group 5 (REM + AP18): HCCs in the group were incubated with 2.5 µM remifentanil for 30 min, followed by incubation with AP18 (TRPA1 channel antagonist, 0.1 mM, 30 min).

Group 6 (SEV + REM): HCCs in the group were incubated with 2.5 µM remifentanil for 30 min, followed by 5.1% sevoflurane for 6 h.

Group 7 (SEV + REM + AP18): HCCs in the group were incubated with 2.5 µM remifentanil for 30 min and then incubated with 5.1% sevoflurane for 6 h, followed by incubation with 0.1 mM AP18 (TRPA1 channel antagonist, 0.1 mM, 30 min).

During calcium signalling analysis (Fura-2/AM), cardiomyocytes were stimulated at the 20th cycle with 0.1 mM cinnamaldehyde in the presence of 1.2 mM calcium and a calcium-free buffer in the extracellular environment. For apoptosis, intracellular reactive oxygen species, mitochondrial depolarisation, caspase-3, and caspase-9 experiments, the HCCs were further treated with the TRPA1 channel agonist cinnamaldehyde (CIN, 0.1 mM, 10 min) to activate the TRPA1 channel before the related analysis.

**Measurement of intracellular free calcium concentration ([Ca^2+^]i)**

Intracellular Ca^2+^ levels were measured using the UV-excitable calcium indicator Fura-2 acetoxymethyl ester (Fura-2-AM). Fluorescence emission at 510 nm was recorded at alternating excitation wavelengths of 340/380 nm every 3 s using a microplate reader (Synergy™ H1, BioTek, USA) following stimulation with cinnamaldehyde (CIN, 0.1 mM). Measurements were performed as previously described. The experiments were conducted by combining the methods described in the studies of Uğuz and Martínez [8,9].

**Apoptosis and intracellular ROS production**

Apoptosis was assessed using the APOPercentage™ assay (Biocolor Ltd., Belfast, Ireland) according to the manufacturer’s instructions. This dye-uptake assay selectively stains apoptotic cells by detecting phosphatidylserine externalisation. Following treatment, apoptotic human cardiomyocytes were quantified spectrophotometrically at 550 nm using a microplate reader (Synergy™ H1, BioTek, Winooski, VT, USA) as described previously [10,11]. Intracellular ROS production was evaluated using Rhodamine 123 (Rh123). Fluorescence intensities were measured at excitation/emission wavelengths of 488/543 nm using the same microplate reader [10,11].

**Caspase-3 and caspase-9 activity assays**

Caspase-3 and caspase-9 activities were determined using fluorogenic substrates Ac-DEVD-AMC and Ac-LEHD-AMC, respectively, as previously reported [10,11]. Fluorescence was measured at 360 nm excitation and 460 nm emission using a microplate reader (Synergy™ H1, BioTek, USA).

**Mitochondrial membrane potential (JC-1) analysis**

Mitochondrial membrane potential (ΔΨm) was assessed using the JC-1 dye (1 µM). Cardiomyocyte cells were incubated with JC-1 at 37 °C for 45 min. Green fluorescence was measured at 485/535 nm and red fluorescence at 540/590 nm using a fluorescence spectrophotometer (Synergy™ H1, BioTek, USA). Mitochondrial depolarisation was expressed as the red/green fluorescence ratio (590/535) and quantified relative to control values [10,11].

**Statistical Analyses**

All results are expressed as means ± standard deviation (SD). Normality of data distribution was assessed, and homogeneity of variances was evaluated using both Bartlett’s and Brown–Forsythe tests. Statistical significance among the groups was determined using one-way analysis of variance (ANOVA), followed by Tukey’s post hoc test for multiple comparisons. Statistical analyses were performed using GraphPad Prism version 7.04 for Windows (GraphPad Software, San Diego, CA, USA). A *p*-value < 0.05 was considered statistically significant, corresponding to a 95% confidence interval.

## 3. Results

**Effects of Sevoflurane and Remifentanil on Cytosolic Calcium Levels in Human Cardiomyocyte Cells (HCCs)**

**Figure 1:**
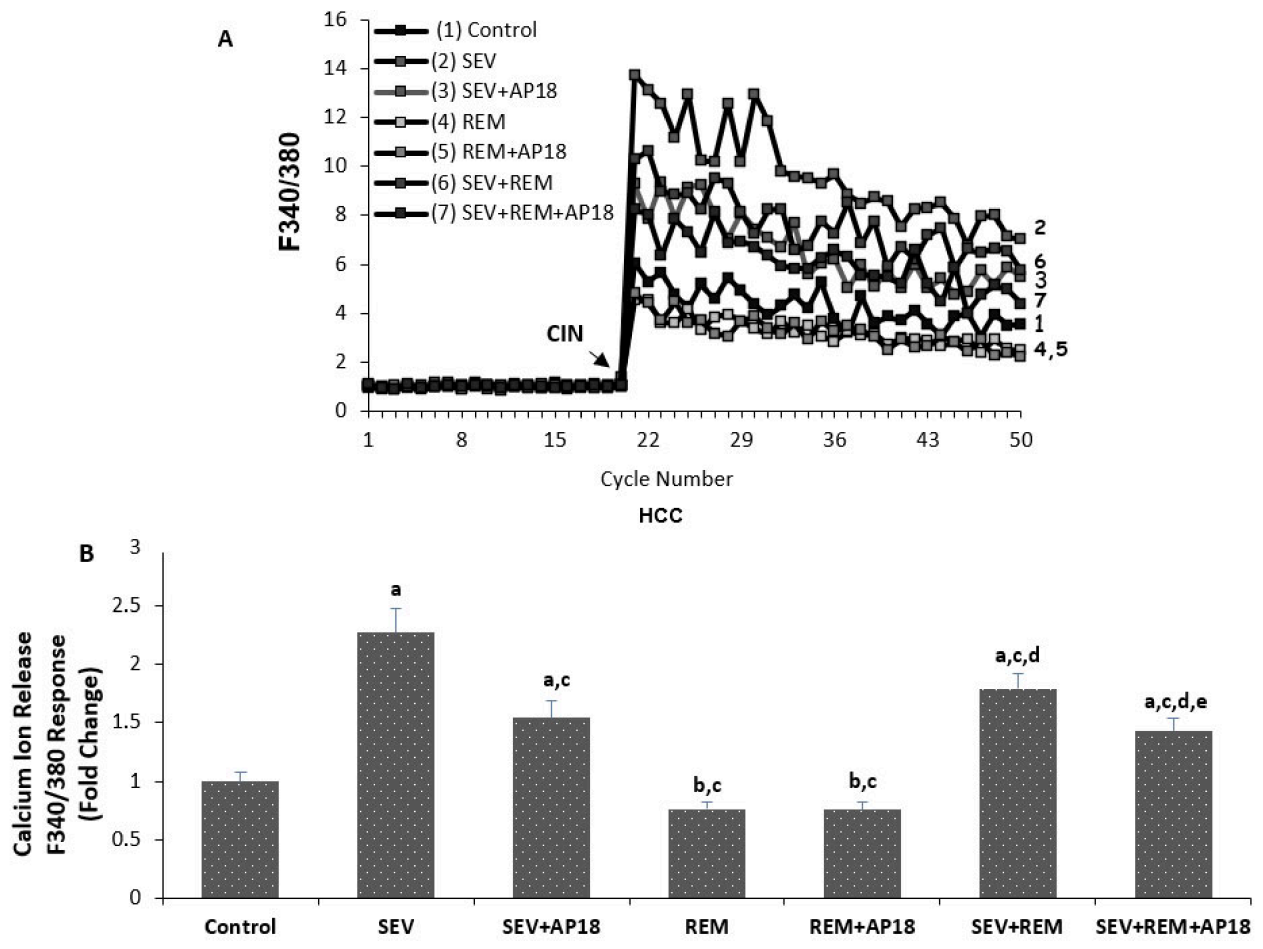
(**A**,**B**) The effect of sevoflurane (SEV, 5.1%, 6 h) and remifentanil (REM, 2.5 µM, 30 min) on cytosolic calcium levels in cardiomyocyte cells. Cells were stimulated by cinnamaldehyde (CIN, 0.1 mM, on the 20th cycle) but were inhibited by AP18 (0.1 mM for 30 min) (mean ± SD, *n* = 3). ^a^ *p* ˂ 0.001 and ^b^ *p* ˂ 0.05 vs. control, ^c^ *p* ˂ 0.001 vs. SEV, ^d^ *p* ˂ 0.001 vs. REM, and ^e^ *p* ˂ 0.05 vs. SEV + REM group.

The effect of sevoflurane and remifentanil administrations on cytosolic calcium levels in HCCs is shown in Figure 1A,B. The TRP Ankyrin 1 channel stimulator cinnamaldehyde and the blocker AP18 were used to evaluate intracellular Ca^2+^ increases through TRPA1 channels in human cardiomyocytes. As shown in Figure 1B, the Ca^2+^ concentration in HCCs was greater in the sevoflurane and sevoflurane + remifentanil groups (*p* < 0.001). It was lower in the remifentanil group than in the control group (*p* < 0.05).

The Ca^2+^ level was lower in the sevoflurane + remifentanil and remifentanil groups in comparison to the sevoflurane group (*p* < 0.001), but at the same time, the remifentanil group was lower than sevoflurane + remifentanil (*p* < 0.001). When sevoflurane was compared with sevoflurane + AP18, and sevoflurane + remifentanil was compared with sevoflurane + remifentanil + AP18, the groups using the AP18 channel inhibitor had lower cytosolic calcium levels than the groups not using AP18 (*p* < 0.001 and *p* < 0.05), and there was no statistically significant difference between the remifentanil and remifentanil + AP18 groups.

**Effects of Sevoflurane and Remifentanil on Programmed Cell Death and Intracellular Reactive Oxygen Species (ROS) and Mitochondrial Depolarisation Levels in Human Cardiomyocyte Cells (HCCs)**

**Figure 2:**
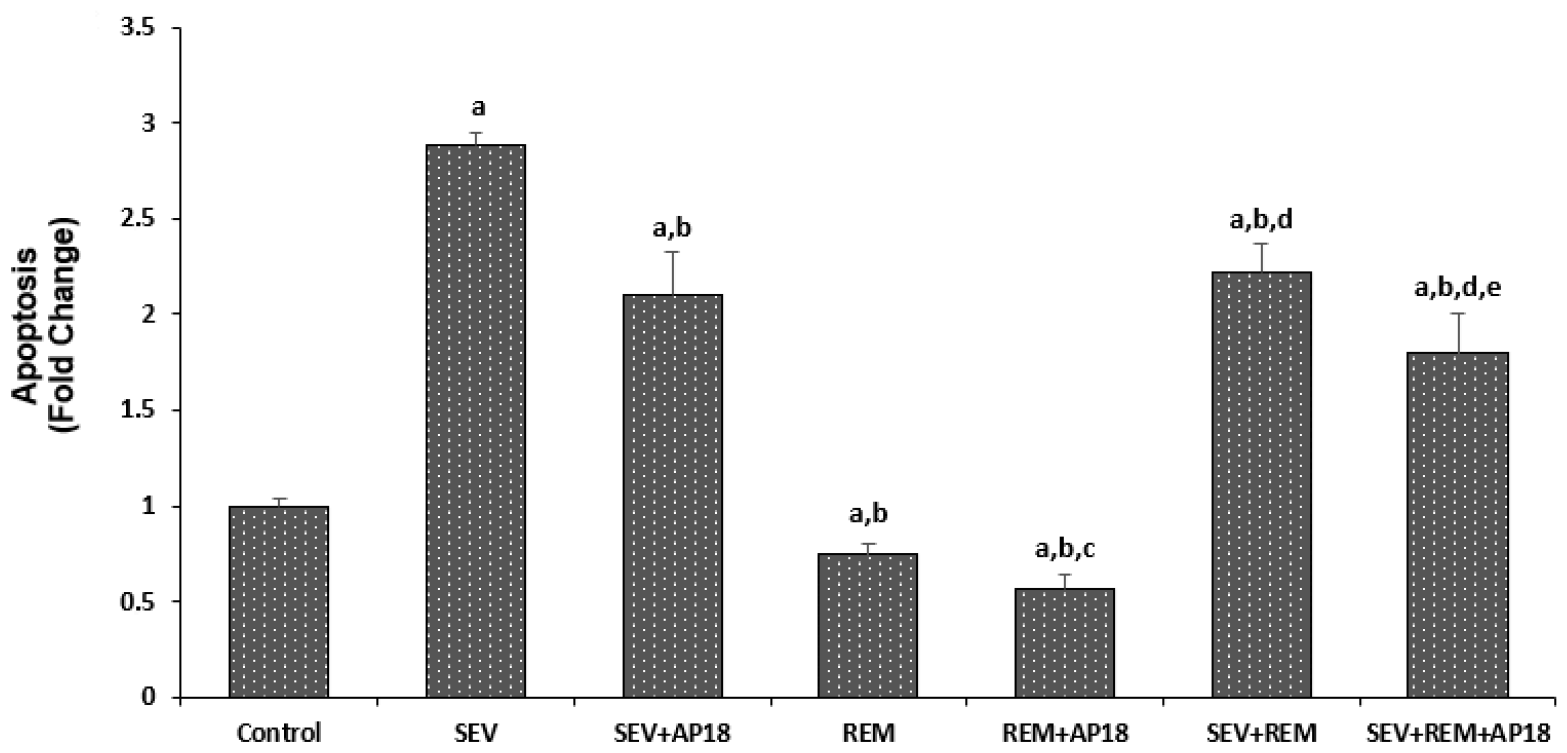
The effect of sevoflurane (SEV, 5.1%, 6 h) and remifentanil (REM, 2.5 µM, 30 min) on apoptosis levels in cardiomyocyte cells. Cells were stimulated by cinnamaldehyde (CIN, 0.1 mM for 10 min) but were inhibited by AP18 (0.1 mM for 30 min) (mean ± SD, *n* = 10). ^a^ *p* ˂ 0.001 vs. control, ^b^ *p* ˂ 0.001 vs. SEV, ^c^ *p* ˂ 0.005 and ^d^ *p* ˂ 0.001 vs. REM, and ^e^ *p* ˂ 0.05 vs. SEV + REM.

**Figure 3:**
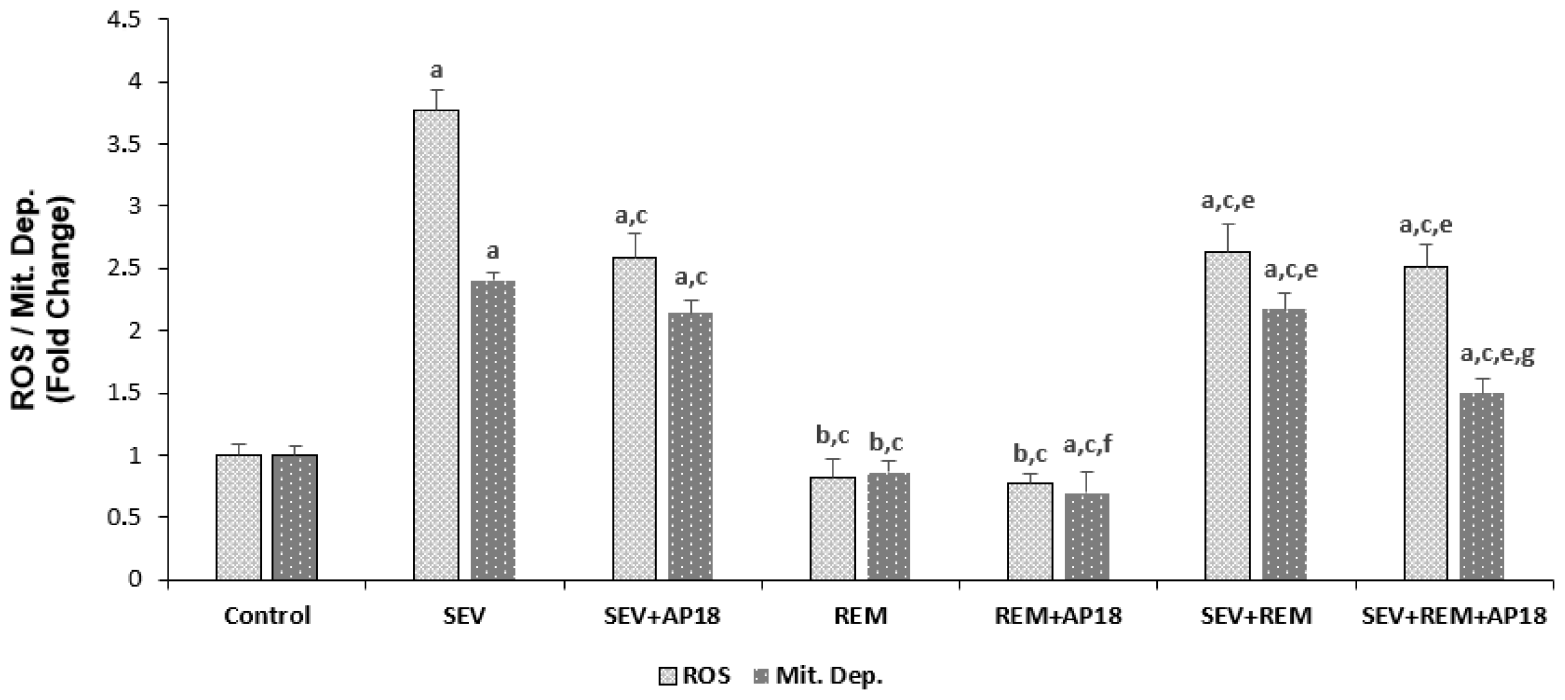
The effect of sevoflurane (SEV, 5.1%, 6 h) and remifentanil (REM, 2.5 µM, 30 min) on reactive oxygen and mitochondrial depolarisation levels in cardiomyocyte cells. Cells were stimulated by cinnamaldehyde (CIN, 0.1 mM for 10 min) but were inhibited by AP18 (0.1 mM for 30 min) (mean ± SD, *n* = 10). ^a^ *p* ˂ 0.001 and ^b^ *p* ˂ 0.05 vs. control, ^c^ *p* ˂ 0.001 vs. SEV, ^e^ *p* ˂ 0.001 and ^f^ *p* ˂ 0.05 vs. REM, and ^g^ *p* ˂ 0.001 vs. SEV + REM.

The effects of sevoflurane and remifentanil administration on apoptosis, ROS levels, and mitochondrial depolarisation in HCCs are shown in Figure 2 and Figure 3. Apoptosis, ROS, and mitochondrial depolarisation levels were higher in the sevoflurane and sevoflurane + remifentanil groups (*p* < 0.001) and lower in all groups in the remifentanil group compared to the control group (*p* < 0.05 and *p* < 0.001). Programmed cell death, ROS, and mitochondrial depolarisation levels were lower in the sevoflurane + remifentanil and remifentanil groups compared with the sevoflurane group (*p* < 0.001). But at the same time, the remifentanil group was lower than sevoflurane + remifentanil when the sevoflurane + remifentanil and remifentanil groups were compared (*p* < 0.001). When sevoflurane was compared with sevoflurane + AP18 and sevoflurane + remifentanil was compared with sevoflurane + remifentanil + AP18, the groups using the AP18 channel inhibitor had lower apoptosis, mitochondrial depolarisation, and ROS levels than the groups not using AP18 (*p* < 0.001 and *p* < 0.05). When remifentanil groups were compared with remifentanil + AP18 groups, the groups using the AP18 channel inhibitor had lower apoptosis and mitochondrial depolarisation levels than the groups without AP18 (*p* < 0.05). Still, there was no statistically significant difference in ROS levels between the remifentanil and remifentanil + AP18 groups.

**Effects of Sevoflurane and Remifentanil on Caspase-3 and Caspase-9 Levels in Human Cardiomyocyte Cells (HCCs)**

**Figure 4:**
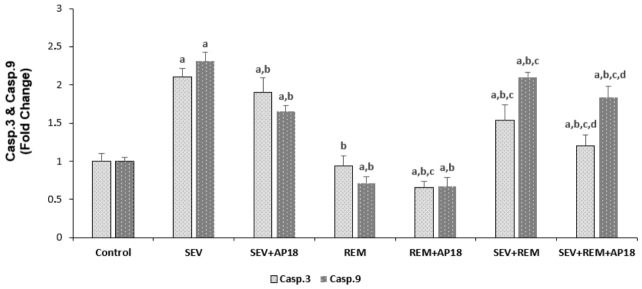
The effect of sevoflurane (SEV, 5.1%, 6 h) and remifentanil (REM, 2.5 µM, 30 min) on caspase-3 and caspase-9 levels in the cardiomyocyte cells. Cells were stimulated by cinnamaldehyde (CIN, 0.1 mM for 10 min) but were inhibited by AP18 (0.1 mM for 30 min) (mean ± SD, *n* = 10). ^a^ *p* ˂ 0.001 vs. control, ^b^ *p* ˂ 0.001 vs. SEV, ^c^ *p* ˂ 0.001 vs. REM, and ^d^ *p* ˂ 0.001 vs. SEV + REM.

The effects of sevoflurane and remifentanil administration on caspase-3 and caspase-9 levels in HCCs are shown in Figure 4. In caspase-3 and caspase-9 analysis, the sevoflurane and sevoflurane + remifentanil groups were greater (*p* < 0.001), and the remifentanil groups were lower in comparison to the control group in caspase-9 analysis (*p* < 0.05). There was no statistically significant difference in caspase-3 analysis. Caspase-3 and caspase-9 levels were lower in the sevoflurane + remifentanil and remifentanil groups in comparison to the sevoflurane group (*p* < 0.001). But at the same time, the remifentanil group was lower than sevoflurane + remifentanil when the sevoflurane + remifentanil and remifentanil groups were compared (*p* < 0.001). When sevoflurane was compared with sevoflurane + AP18, and sevoflurane + remifentanil was compared with sevoflurane + remifentanil + AP18, the groups using the AP18 channel inhibitor had lower levels of caspase-3 and caspase-9 than the groups not using AP18 (*p* < 0.001). When remifentanil was compared with the remifentanil + AP18 group, the AP18 channel inhibitor group had lower caspase-3 levels than the non-AP18 group (*p* < 0.001). Still, there was no statistically significant difference between the remifentanil and remifentanil + AP18 groups in caspase-9 analysis.

## 4. Discussion

The present study demonstrates that exposure to sevoflurane induces apoptotic damage in human cardiomyocytes and that pre-treatment with remifentanil significantly attenuates this apoptotic effect. These results are consistent with the existing literature and provide novel insights into the potential role of TRPA1 calcium channels in this process. The observation that sevoflurane triggers apoptosis in human cardiomyocytes aligns with previous studies examining the cytotoxicity of this volatile anaesthetic. Piao et al. demonstrated that sevoflurane exposure induces neuronal cell death through parthanatos, a PARP-1-dependent programmed cell death pathway [1]. Our findings extend this concept to the cardiovascular system, suggesting that sevoflurane may similarly exert pro-apoptotic effects in human cardiomyocytes, likely through oxidative stress mechanisms. This is particularly relevant given the high metabolic activity and cardiac tissue’s vulnerability to oxidative damage. Moreover, Yang et al. [5] reported that inhibition of another TRP channel, TRPV1, protects against sevoflurane-induced cell death, indicating a broader role for TRP channels in anaesthetic-induced cytotoxicity. The current study builds upon these observations by focusing specifically on TRPA1 as a candidate mediator in human cardiomyocytes.

TRPA1 channels as mediators of sevoflurane-induced apoptosis; The involvement of TRPA1 in our model is supported by several complementary lines of evidence: (i) sevoflurane increased intracellular Ca^2+^, an effect reduced by the TRPA1 antagonist AP18; (ii) AP18 also attenuated sevoflurane-induced apoptosis, ROS production, mitochondrial depolarisation, and caspase-3/-9 activities; and (iii) the TRPA1 agonist cinnamaldehyde elicited Ca^2+^ responses, confirming functional expression of the channel in human cardiomyocytes. These results align with studies demonstrating a pro-apoptotic role for TRPA1 in cardiac settings. Li et al. [12] found that TRPA1 inhibition promotes cardiac repair after infarction by reducing apoptosis and fibrosis. Wang et al. showed that remifentanil reduces oxidative stress, inflammation, and apoptosis, thereby maintaining mitochondrial biogenesis in sepsis-associated cardiotoxicity [13]. At the same time, Su and Chen [14] reported that TRPA1 blockade attenuates doxorubicin-induced cardiotoxicity through suppression of oxidative stress and ER stress. Conversely, some reports have suggested that TRPA1 activation may be protective under certain conditions [15], indicating that the outcome of TRPA1 signalling is context-dependent. Our data suggest that, in the face of sevoflurane-induced oxidative stress, excessive TRPA1 opening leads to calcium overload, mitochondrial depolarisation, and initiation of the intrinsic apoptotic cascade. This is consistent with ROS’s known ability to directly activate TRPA1 via covalent modification of cysteine residues, creating a feed-forward loop that amplifies cell damage. It is also worth comparing our findings with those of Yang et al. [5], who demonstrated that inhibition of another TRP channel, TRPV1, protects against sevoflurane-induced cell death. Although both TRPV1 and TRPA1 are Ca^2+^-permeable and responsive to oxidative stress, their distinct activation mechanisms and downstream effectors may explain why targeting different TRP channels can be protective. Our study specifically implicates TRPA1, providing a more focused mechanistic insight into sevoflurane cardiotoxicity.

The channel’s known properties support the hypothesis that sevoflurane-induced ROS production activates TRPA1 channels. TRPA1 is a non-selective cation channel with high calcium permeability that is directly activated by reactive oxygen and carbonyl species. Li et al. (2020) found that inhibition of TRPA1 promotes cardiac repair after myocardial infarction by reducing cardiac fibrosis and cell apoptosis and activating the PI3K/Akt signalling pathway [6]. While Li et al. focused on ischaemia models, their results indicate a pathological role for TRPA1 activation in the heart, consistent with our hypothesis that sevoflurane-mediated TRPA1 opening could lead to calcium overload and subsequent apoptosis. Further supporting a pro-apoptotic role for TRPA1, studies in chondrocytes have shown that TRPA1 mediates IL-1β-induced apoptosis via calcium overload and mitochondrial dysfunction [14]. Conversely, Andrei et al. (2019) reported that TRPA1 stimulation with AITC attenuated ischaemia-induced cardiomyocyte cell death through an eNOS-dependent mechanism [7], suggesting a context-dependent role for TRPA1 activation [15]. These divergent findings highlight the complexity of TRPA1 signalling, which may depend on stimulus nature and duration, cell type, and the presence of cofactors. In the context of sevoflurane exposure, our working model posits that excessive ROS production leads to aberrant TRPA1 activation and calcium influx, tipping the balance toward cell death rather than survival.

Our finding that remifentanil pretreatment reduces sevoflurane-induced apoptosis is strongly supported by a substantial body of evidence demonstrating its cardioprotective properties. A comprehensive review by Zhu et al. (2023) highlights that remifentanil inhibits calcium overload, inflammation, oxidative stress, and apoptosis, thereby conferring protection against ischaemia–reperfusion (I/R) injury in multiple organs, including the heart [8]. Dou and colleagues specifically showed that remifentanil preconditioning protects rat cardiomyocytes against hypoxia–reoxygenation injury via δ-opioid receptor-mediated activation of the PI3K/Akt and ERK pathways [2]. These signalling cascades are well-established pro-survival pathways that can counteract apoptotic stimuli. Kim et al. (2010) also reported that remifentanil reduces myocardial infarct size and preserves anti-apoptotic proteins (Bcl-2) while reducing pro-apoptotic proteins (Bax and cytochrome c) in I/R rat hearts, regardless of the timing of administration [9,12]. Chen et al. extended these findings by demonstrating that remifentanil postconditioning protects H9c2 cardiomyoblasts from hypoxia/reoxygenation injury by inhibiting endoplasmic reticulum stress-induced apoptosis [10]. Additionally, Lewinska et al. showed for the first time that remifentanil preconditioning protects human cardiomyocytes from hypoxia-induced cellular senescence and necroptosis [11], further emphasising its broad cytoprotective potential in human cardiac cells. Remifentanil has also been shown to reduce oxidative stress, inflammation, and apoptosis in sepsis-associated cardiotoxicity by inhibiting NF-kB and caspase-3 while enhancing SIRT1 expression [13], indicating its ability to modulate key stress response pathways.

A key novel aspect of the current study is the investigation of whether remifentanil might exert its protective effects, at least in part, by modulating TRPA1 channel activity. Direct evidence linking remifentanil to TRPA1 is currently sparse, but several lines of indirect evidence support this possibility. Given that TRPA1 can be activated by oxidative stress, the ability of remifentanil to reduce ROS production—as demonstrated in various models—could, in turn, decrease TRPA1 activation. Furthermore, remifentanil’s inhibition of calcium overload could mitigate the downstream consequences of TRPA1-mediated calcium influx. It is also plausible that remifentanil could modulate TRPA1 channel gating, either directly or indirectly, possibly through opioid receptor signalling pathways that intersect with TRP channel regulation. The PI3K/Akt pathway, activated by remifentanil [3], lies downstream of TRPA1 in some contexts, suggesting a complex interplay that warrants further investigation. Alternatively, the lack of functional TRPA1 expression in cardiomyocytes has been debated, with some studies questioning its direct presence in these cells. However, other research has demonstrated functional TRPA1 expression in adult mouse cardiac tissue and its role in modulating contractility and cell survival [6,7]. Regardless of its exact cellular localisation, TRPA1 in other cardiac cell types (e.g., endothelial cells and fibroblasts) could indirectly influence cardiomyocyte fate.

The sevoflurane-induced increase in cytosolic Ca^2+^ observed in our study is a critical upstream event. Excessive Ca^2+^ uptake by mitochondria triggers opening of the mitochondrial permeability transition pore, dissipates the membrane potential, and releases pro-apoptotic factors such as cytochrome c, which in turn activate caspase-9 and downstream caspase-3 [10,11]. Our data showing increased caspase-9 and -3 activities, along with JC-1-detected depolarisation, are fully consistent with this canonical intrinsic apoptotic pathway. Remifentanil’s ability to blunt the Ca^2+^ rise and preserve mitochondrial function likely interrupts this cascade at an early stage. The fact that AP18 partially reversed remifentanil’s effects on mitochondrial depolarisation and caspase activities further supports the notion that TRPA1-mediated Ca^2+^ entry is a key node in this pathway. However, the incomplete reversal also implies that remifentanil may stabilise mitochondrial function through additional mechanisms, such as direct inhibition of mPTP opening or enhancement of mitochondrial biogenesis [13].

The current findings have potential clinical implications for perioperative myocardial protection. Sevoflurane and remifentanil are frequently co-administered during cardiac and non-cardiac surgery. While sevoflurane has well-documented cardioprotective effects under certain conditions (e.g., ischaemic preconditioning) [16,17], our data suggest that, under direct exposure without additional protective stimuli, it may exert pro-apoptotic effects that can be mitigated by remifentanil. This aligns with the concept of balanced anaesthesia, in which the combination of agents may offer synergistic benefits beyond their primary pharmacological actions. Clinical studies have shown that sevoflurane combined with remifentanil effectively maintains vital signs in patients with coronary heart disease undergoing non-cardiac surgery, protecting cardiomyocytes and reducing cardiovascular events. However, the balance between sevoflurane’s protective and toxic effects likely depends on concentration, duration of exposure, and the presence of comorbidities such as diabetes or ischaemia, which can influence remifentanil’s efficacy [18]. Furthermore, the role of TRPA1 in the human heart remains to be fully elucidated, and inter-individual variability in TRPA1 expression or function could influence the protective effect of remifentanil.

The co-administration of sevoflurane and remifentanil is routine in cardiac anaesthesia. Our findings suggest that remifentanil may serve not only as an analgesic but also as a cytoprotective adjuvant that counteracts the potential pro-apoptotic effects of sevoflurane. This is particularly relevant for patients with conditions that predispose them to oxidative stress, such as diabetes or ischaemic heart disease, where the efficacy of remifentanil may be altered [18]. However, several caveats must be considered before direct clinical translation. The concentration of remifentanil used (2.5 µM) is higher than typical plasma levels; nevertheless, tissue concentrations may be higher, and the in vitro setting necessitates such levels to achieve detectable effects within a short timeframe. Moreover, our in vitro model lacks the neurohumoral, inflammatory, and haemodynamic influences present in vivo, which could modify the observed responses. Future studies using animal models or human cardiac tissue, combined with additional specific TRPA1 inhibitors or siRNA knockdown, would help to confirm the mechanistic role of TRPA1 and to establish the optimal dosing and timing of remifentanil for myocardial protection.

## 5. Limitations and Future Directions

Several limitations warrant consideration when interpreting the present findings. First, while the in vitro human cardiomyocyte model enables controlled mechanistic studies, it inherently lacks the complex in vivo perioperative milieu, including neurohumoral factors, inflammatory cells, and haemodynamic forces. Consequently, the observed cellular and molecular responses may not fully reflect physiological interactions, and our observations should be considered preliminary with limited direct extrapolation to clinical practice. Second, the proposed role of TRPA1 in sevoflurane-induced cardiomyocyte injury and remifentanil-mediated protection was inferred primarily through pharmacological modulation with the agonist cinnamaldehyde and the antagonist AP18, along with assessments of intracellular calcium, reactive oxygen species production, mitochondrial membrane depolarisation, and caspase-9/caspase-3 activation. While these data consistently support TRPA1-associated calcium signalling, they do not definitively establish a causal relationship. Future investigations should employ selective TRPA1 antagonists (e.g., HC-030031), siRNA-mediated knockdown, or knockout models to confirm this role.

Furthermore, quantitative evaluations of TRPA1 mRNA and protein expression, as well as electrophysiological recordings, are necessary to provide definitive mechanistic validation. Third, the specific downstream signalling cascades—particularly PI3K/Akt, ERK, and p38 MAPK—through which remifentanil modulates TRPA1 activity or its downstream apoptotic effects require detailed elucidation. Fourth, the remifentanil concentrations applied (0.1–10 μM) are considerably higher than clinically relevant plasma levels, which typically fall in the low nanomolar range. This discrepancy necessitates careful interpretation, and potential off-target effects of remifentanil on other TRP channels or calcium-handling proteins should be systematically explored. Regarding methodological endpoints, although our fluorescence-based assays are well-validated and widely accepted, we acknowledge that incorporating flow cytometry would offer enhanced quantitative resolution, particularly in distinguishing early versus late apoptotic populations.

Additionally, while we assessed multiple biochemical and cellular endpoints, we did not evaluate functional cardiomyocyte parameters such as contractility or spontaneous beating activity. Including these functional analyses in future work would substantially strengthen the physiological relevance of our molecular findings. Finally, given the inherent limitations of in vitro models and the preliminary nature of our pharmacological data, well-designed animal studies and rigorous clinical investigations are essential to validate the translational and clinical significance of the proposed protective signalling pathway.

## 6. Conclusions

In conclusion, the present study demonstrates that sevoflurane induces apoptosis in human cardiomyocytes, an effect that is significantly mitigated by remifentanil pretreatment. The protective action of remifentanil is associated with reduced oxidative stress and preserved mitochondrial membrane potential, highlighting its role in counteracting the pro-apoptotic cascade triggered by the volatile anaesthetic. Critically, the partial reversal of remifentanil-mediated protection by the TRPA1 antagonist AP18 suggests that TRPA1 calcium channels contribute to this process, potentially linking sevoflurane-induced oxidative stress to apoptotic cell death and identifying a novel target through which remifentanil may exert its cytoprotective effects. However, the incomplete reversal also implies that additional, TRPA1-independent mechanisms are likely involved. These findings are consistent with the existing literature on the cardioprotective properties of remifentanil and the pro-apoptotic potential of sevoflurane, while adding a novel mechanistic perspective. Collectively, our results hold potential implications for perioperative myocardial protection during combined sevoflurane–remifentanil anaesthesia. Nevertheless, further mechanistic and translational investigations are warranted to fully elucidate these signalling pathways and determine the clinical relevance of TRPA1 modulation in the perioperative setting.

## Data Availability

The original contributions presented in this study are included in the article. Further inquiries can be directed to the corresponding author.
